# Plasma Biomarkers of Alzheimer’s Disease and Neurodegeneration According to Sociodemographic Characteristics and Chronic Health Conditions

**DOI:** 10.14283/jpad.2024.142

**Published:** 2024-07-14

**Authors:** H. T. Zheng, Z. Wu, M. M. Mielke, A. M. Murray, Joanne Ryan

**Affiliations:** 1 School of Public Health and Preventive Medicine, Monash University, Melbourne, VIC Australia; 2 Department of Epidemiology and Prevention, Wake Forest University School of Medicine, Winston-Salem, NC USA; 3 Division of Geriatric and Palliative Medicine, Department of Medicine, Hennepin Healthcare, Minneapolis, Minnesota USA; 4 Berman Center for Outcomes and Clinical Research, Hennepin Healthcare Research Institute, Minneapolis, Minnesota USA

**Keywords:** Alzheimer’s disease, neurodegeneration, amyloid, tau, plasma biomarkers

## Abstract

Ultrasensitive assays have been developed which enable biomarkers of Alzheimer’s disease pathology and neurodegeneration to be measured in blood. These biomarkers can aid in diagnosis, and have been used to predict risk of cognitive decline and Alzheimer’s disease. The ease and cost-effectiveness of blood collections means that these biomarkers could be applied more broadly in population-based screening, however it is critical to first understand what other factors could affect blood biomarker levels. The aim of this review was to determine the extent that sociodemographic, lifestyle and health factors have been associated with blood biomarkers of Alzheimer’s disease and neuropathology. Of the 32 studies included in this review, all but one measured biomarker levels in plasma, and age and sex were the most commonly investigated factors. The most consistent significant findings were a positive association between age and neurofilament light chain (NfL) and glial fibrillary acidic protein (GFAP), and females had higher GFAP than men. Apolipoprotein ε4 allele carriers had lower Aβ42 and Aβ42/40 ratio. Body mass index was negatively associated with GFAP and NfL, and chronic kidney disease with higher levels of all biomarkers. Too few studies have investigated other chronic health conditions and this requires further investigation. Given the potential for plasma biomarkers to enhance Alzheimer’s disease diagnosis in primary care, it is important to understand how to interpret the biomarkers in light of factors that physiologically impact blood biomarker levels. This information will be critical for the establishment of reference ranges and thus the correct interpretation of these biomarkers in clinical screening.

## Introduction

**T**he pathway to receiving a diagnosis of Alzheimer’s disease (AD) can be long and complex, involving detailed medical assessments, laboratory tests, cognitive assessments, and neurological examinations (1, 2). A diagnosis of AD currently relies on positron emission tomography (PET) imaging or measurement of amyloid beta (Aβ) and phosphorylated Tau (p-tau) in cerebrospinal fluid (CSF) (3, 4). However, the high cost, invasiveness and relatively low availability of these specialized tools has prevented their widespread use (4). Furthermore, many individuals only receive a dementia diagnosis at an advanced stage of the disease, despite the fact that deposition of amyloid plaques and neurofibrillary tangles commences years prior to the onset of clinical symptoms (2). Early detection of AD pathology would enable timely treatments, including recruitment into clinical trials, tracking progression of the disease, and even early interventions for individuals at high risk of AD before the onset of symptoms.

Ultrasensitive assays have now been developed which permit Aβ and p-tau to be measured in blood. The findings from multiple studies have demonstrated the utility of these biomarkers to aid in the diagnosis of AD (5, 6) and to predict cognitive decline (7, 8) and the risk of AD. The ratio of Aβ42/40 appears to be a surrogate biomarker of cortical Aβ deposition, independent of clinical AD diagnosis (9, 10). Plasma p-tau 181, and p-tau 217 show high concordance with levels of brain amyloid (based on PET imaging) (9, 11), and have excellent diagnostic performance for differentiating individuals with AD from other forms of dementia (12, 13). Neurofilament light chain (NfL), a non-specific marker of neuronal injury (14, 15) has been associated with the risk of AD, vascular dementia, and other types of dementia (e.g. Frontal temporal lobe dementia (8, 16, 17). Glial fibrillary acidic protein (GFAP) is a marker of astroglial activation and neuroinflammation. Plasma GFAP levels correlate with amyloid accumulation and are higher among individuals with versus without preclinical AD pathology (18, 19). Combining these blood-based biomarker measures can also provide additional insight (20). These biomarkers may thus provide an accurate, more cost-effective and timely way to diagnose AD at the population level than CSF or PET scans, and contribute to monitoring of disease progression.

Given that these blood AD biomarkers are now available for clinical use, it is critical to better understand what factors could influence levels of these biomarkers. Some factors like the apolipoprotein E allele (APOE-ε4), the strongest genetic risk factor for AD, may affect blood biomarkers levels due to its direct impact on amyloid pathology (21). On the other hand, hepatic and renal function could modify blood biomarker concentrations through impaired peripheral clearance (22) independent of a direct link with AD (23). Indeed, a range of factors could potentially affect blood biomarker levels, as a reflection of their status as risk factors for AD, via their influence on other physiological processes, or both. Understanding the impact of these factors on the blood biomarker levels is essential for correctly interpreting the blood biomarkers for the diagnosis and prognosis of AD. The aim of this review was to determine which sociodemographic, lifestyle and health factors have been associated with blood biomarkers of AD pathology.

## Method

Given the broad aim to summarise the evidence for a range of factors which may be associated with blood biomarker levels, a narrative review was undertaken. Literature were retrieved from electronic databases (e.g. PubMed, Embase and Google Scholar) up until 20th March 2024, using search terms: (biomarker*) AND (blood or plasma or serum) AND (dement* or Alzheimer* or MCI or cogniti*). The literature search was limited to human studies and articles published in English. Snowball sampling was also used (i.e. identifying studies from the reference lists of relevant articles). Authors TZ, ZU and JR assessed the eligibility of studies for inclusion.

### Inclusion Criteria

Studies eligible for inclusion reported blood biomarkers Aβ42, Aβ40, the ratio of Aβ42 to Aβ40 (Aβ42/40), p-tau (e.g. 181, 217), NfL and/or GFAP, and investigated whether these biomarker levels varied according to participant socio-demographic characteristics, lifestyle factors and chronic health conditions. More specifically, we included studies that measured plasma or serum using an immunoassay or Mass Spectroscopy based platform (e.g. SIMOA, Roche elecsys, Lumipulse, IP-MS). Studies measuring t-tau were also included, although it is recognised that most current studies are no longer measuring this protein. With regards to factors that may affect biomarker levels, we focused on chronic health conditions (e.g., chronic kidney disease, diabetes, dyslipidaemia), lifestyle factors (e.g. alcohol consumption, smoking, exercise) as well as sociodemographic factors (e.g., age, sex, ethnicity and race), and body mass index (BMI). We included studies of participants across all age groups and the clinical disease spectrum with diverse characteristics and health conditions.

### Exclusion Criteria

We excluded studies that only measured AD biomarkers in other biofluids (e.g. CSF), or studies that only focused on other blood biomarkers. We also excluded studies that only reported on the association between these biomarkers and cognition and/or dementia, but did not specifically report on the impact of sociodemographic or chronic conditions on the blood biomarker levels. Studies that only examined whether these biomarkers could predict future risk of disease (e.g. incident heart failure) were also not eligible for inclusion. Reviews, and abstracts published from conferences, were not included.

### Data Extraction

A predesigned data extraction form, created specifically for this review, was used. For each study the following information was retrieved: study design, name (if applicable) and country in which it was conducted; basic characteristics of the study participants, namely age, sex, frequency of APOE-ε4 allele (if reported), and whether the participants had cognitive impairment or dementia; the socio-demographic and health factors examined; the blood biomarkers examined; and the main findings. TZ extracted all of the data, and this information was checked by JR and ZW.

### Data Synthesis

As this is a narrative review, a meta-analysis could not be performed, and risk of bias assessment was not undertaken. The results of the included studies were instead summarised qualitatively.

## Results

Table 1 shows a summary of the 32 studies which were included in this review (18, 22, 24–48). These were predominantly cohort studies conducted in the US and Canada (18 studies) or Europe (9 studies), but some were also conducted in China (3 studies) and Australia (4 studies). The size of the studies varied from 92 (less than 100) to 4444 (over 4000), with only a quarter (8 studies) having over 1000 participants. Sixteen studies examined p-tau (p-tau 181, 217), 19 examined Aβ (Aβ40, Aβ42 and Aβ42/40 ratio), 10 GFAP and 20 NfL. Eleven studies measured t-tau, and given that this biomarker is no longer commonly used, the results here will focus on the other biomarkers. All but five studies measured plasma biomarkers using the Simoa platform, and only one study measured biomarkers in serum (31).

**Table 1:** Summary of studies reporting an association between blood AD and neurodegeneration biomarkers and sociodemographic, lifestyle or health factors

**Ref.**	**Study (Country)**	**n: mean age (SD) or range, sex, ethnicity (if given)**	**Factor(s)**	**Blood Biomarker**	**Main finding**
Wang et al., 2017 (24)	Hospital patients (China)	Total 92 (46 cases, 46 control) Cases: age: 54.22 (1.57), 28.9% ♀, Controls: age: age: 52.46(1.54), 28.3% ♀	Cirrhosis	Plasma Aβ40, Aβ42 [Simoa]	• Cirrhosis: ↑ Aβ40, Aβ42
Nakamura et al., 2018 (25)	NCGG cohort (Japan) and AIBL (Australia)	121 NCGG: 41.3% Aβ+, age: 74.0±5.1, 54.5% ♀, 41.3% APOE ε4 252 AIBL: 54.4% Aβ+, age: 74.2±5.8, 48.8% ♀,42.9% APOE ε4	Age, sex	Plasma APP669–711, Aβ42, Aβ42/40 ratio [IP-MS]	• Age: NS • Sex: NS
Pase et al., 2019 (26)	Framingham Heart Study (US) & Memento study (France)	Total 1453 Framingham cohort: age 75 (7), 54.5% ♀, 21% APOE ε4	Age, sex, education, APOE ε4	Plasma t-tau [Simoa]	• Age: + t-tau • Sex: female ↑ t-tau • Education: ↓ t-tau • APOE ε4 allele: NS
de Wolf et al., 2020 (27)	Rotterdam study (Netherlands)	Total 4444, age 71.9 (7.5), 58% ♀, 9% MCI, 25.7% APOE ε4	Age	Plasma t-tau, NfL, Aβ40 and Aβ42 [Simoa]	• Age: + NfL & Aβ40, − Aβ42/40
Hu et al., 2020 (28)	Population cohort (China)	Total 1282, 62.1% ♀, age: 55.7 (10.2), 13.5% APOE ε4	Blood lipids	Plasma Aβ42, Aβ40 [ELISA]	• TC: + Aβ42 (normal BP) • LDL-c: + Aβ42 (normal BP), − Aβ40 (high BP)
Brickman et al., 2021 (77)	Washington Heights-Inwood Columbia Aging Project (US)	Total 413. Autosy: 113, age: 85.64, 61% ♀, 29% APOE ε4; Clinical: 300, age: 81.87, 67% ♀, 28% APOE ε4. Multiethnic	Age, sex, APOE ε4, BMI, ethnicity	Plasma Aβ42/40, p-tau181, p-tau217, t-tau, NfL [Simoa]	• Age : + t-tau, p-tau181, p-tau217, NfL • APOE ε4 : ↑ p-tau181, p-tau217 • BMI : ↓ p-tau181, p-tau217, NfL • Sex, ethnicity: NS
Chatterjee et al., 2021 (18)	KARVIAH cohort (Australia)	Total 100, 33% Aβ+, age: 79.0±5.4, 61% ♀, APOE ε4: 48.5%. Aβ−: age: 77.8±5.6, 72% ♀, APOE ε4: 7.5%	Age, sex, APOE ε4,	Plasma NfL, GFAP, t-tau, p-tau181, p-tau231 [Simoa]	• Age: + GFAP, p-tau181, p-tau231 & NfL • Sex: females ↑ t-tau • APOE ε4 allele: ↑ p-tau231
Chatterjee et al., 2021 (29)	KARVIAH cohort (Australia)	Total 96, 34% Aβ+ age: 79.6 ± 5.2, 61% ♀, APOE ε4: 42.4%; Aβ−: age: 77.4 ± 5.5, 71% ♀, APOE ε4: 7.9%	Age, sex, APOE ε4	Plasma Aβ42, Aβ40, Aβ42/40 ratio [Simoa]	• APOE ε4 allele: ↓ Aβ42, Aβ42/ 40 • Sex: NS
Darmanthe et al., 2021 (30)	ADNI (US and Canada)	440 MCI, age: 73.5 (7.6), 41.8% ♀	Age, sex, education	Plasma NfL [Simoa]	• Age: + NfL • Sex & Education: NS
Desai et al., 2021 (31)	Chicago Health and Aging Project (CHAP) (US)	Total 1159, age: 77.4 (6.0), 63% ♀, 60% African American	Physical activity	Serum t-tau [Simoa]	• Physical activity: ↓ t-tau
Kaeser et al., 2021 (32)	Age cohort, Centenarian cohort, Nonagenarian cohort (Denmark)	122 Age cohort, age: 72 (21–107), 51% ♀; 135 Centenarian cohort, age: 100, 79% ♀, 24.5% APOE ε4 180 Nonagenarian cohort, age: 92.9 (92.2–93.7), 76% ♀, 24.3% APOE ε4	Age, sex	Plasma NfL [Simoa]	• Age: + NfL • Sex: NS
Keshavan et al., 2021 (33)	Medical Research Council National Survey for Health and Development (UK)	Total 441 dementia-free, white 359 amyloid PET-negative: n = 359, age: 70.7 (0.7), 49.6 ♀, 22% APOE ε4; 82 amyloid PET-positive: age: 70.6 (0.6), 54.9 ♀, 57.3% APOE ε4	Age, sex	Plasma Aβ40, Aβ42, Aβ42/40, p-tau181 [Simoa]	• Age: + Aβ42, Aβ42/40, p-tau181 • Female ↓ Aβ40
Sullivan et al., 2021 (34)	Atherosclerosis Risk in Communities cohort study (US)	Total 2284, age=59.2±5.2, 57% ♀, 22% Black, 29% APOE ε4	Age, sex, APOE ε4	Plasma Aβ42, Aβ40, Aβ42/40 [INNO-BIA assay]	• Age: + Aβ40 and Aβ42, − Aβ42\40 • Female ↓Aβ42, Aβ40 • APOE ε4 allele: ↓Aβ42
Berry et al., 2022 (22)	Prospective cohorts (US). FALT study (cases); Hillblom Healthy Aging study (controls).	Controls 22. age: 58 (11), 68% ♀, 91% Non-Hispanic white, 9% Asian. Cirrhosis cases 135. Age: 59 (9), 71% ♀, 55% Hispanic, 30% Non-hispanic white, 10% Asian, 5% others	Age, sex, BMI, hypertension, diabetes, cirrhosis severity & sequelae	Plasma p-tau181, NfL, GFAP, t-tau [Simoa]	• Age & stroke: ↑ NfL • Sex, BMI, hypertension, CAD: all NS • Diabetes & Dialysis: ↑ p-tau181 & NfL • Cirrhosis: ↑ p-tau181, NfL, t-tau Cirrhosis severity: • Creatinine: ↑ p-tau181, NfL, t-tau • Albumin : ↑ p-tau181, NfL, t-tau, GFAP • Neurologic injury markers : ↑ NfL
Jiang et al., 2022 (37)	HABS-HD study (US)	Total 1521, age: 66.3 ± 8.7, 61% ♀	Age, sex, APOE, education, lifestyle factors, CVD risk score, depression	Plasma Aβ42/40, t-tau, NfL [Simoa]	• Age: + t-tau, NfL • Females & Mexican American: ↑ Aβ42/40 & t-tau, ↓ NfL • APOE status: ↓ Aβ42/40 • Education: + NfL, – Aβ42/40 & t-tau • High CVD risk: ↑ t-tau, ↑ NfL (MA only) • Physical activity: ↓ t-tau • Depression: ↑ Aβ42/40, t-tau, NfL • Alcohol: NS
Mielke et al., 2022 (38)	MCSA cohort (US)	Total 1329, 26.6% APOE ε4 1161 controls: age: 70.9 (48.4, 79.8), 45.8% ♀, 96.8% white; 153 MCI: age: 80.8 (75.8, 86.1), 42.5% ♀, 97.4% white; 15 dementia: age: 83.5 (81.5, 85.0), 13.3% ♀, 93.3% white	Age, sex p ]BMI, education, multiple p ]comorbidities p ][electrochemiluminescence]	Plasma p-tau181, p-tau217	• Age: + p-tau181 & p-tau217 • Males ↑ p-tau217 (not in MCI/dementia) • APOE ε4 allele, hypertension, CKD, Stroke, MI, head trauma: ↑ p-tau181 & p-tau217 • BMI: - p-tau181 & p-tau217 • Smoking: ↓ p-tau181 & p-tau217 • NS: education, depression, anxiety, cancer, dyslipidemia, diabetes, , atrial fibrillation
O’Bryant et al., 2022 (40)	HABS-HD (US)	Total 965 (all CN): age: 66.75, 65% ♀, edu: 12.88, Mexican American 46%	Ethnicity, health conditions	Plasma Aβ42, Aβ40, Aβ42/40, t-tau, NfL, GFAP [Simoa]	• Ethnicity : MA ↓ Aβ40, ↑ t-tau, ↑ Aβ42/40 • Dyslipidemia: ↑ Aβ40, Aβ42 • Hypertension : ↑ Aβ40, Aβ42, t-tau • Diabetes & CKD: ↑ Aβ40, Aβ42, t-tau, NfL
Pajewski et al., 2022 (39)	Systolic Blood Pressure Intervention Trial (SPRINT) (US)	Total 517. 283 Intensive treatment, age: 69.8 (6.9), 44.5% ♀, 67.8% white, 28.6% Black; 234 Standard treatment, age: 69.9 (7.4), 41% ♀, 65% white, 27.8% Black	Age, eGFR	Plasma Aβ40 and Aβ42, Aβ42/40, t-tau, NfL [Simoa]	• Age: + Aβ40 & Aβ42, t-tau, NfL • eGFR: + Aβ40 and Aβ42, t-tau, NfL
Pichet Binette et al., 2022 (41)	BioFINDER (Sweden)	BioFINDER-1: 748, age: 71.7, 48% ♀ BioFINDER-2: 421, age: 65.7, 50% ♀	BMI, cirrhosis severity, health conditions	Plasma Aβ42/40, p-tau181, p-tau217, NfL, GFAP [Simoa]	• BMI : - NfL, GFAP, p-tau217, p-tau181 • Creatinine : + NfL. GFAP, p-tau 217 (in BioFINDER-1only) • Hypertension, diabetes, dyslipidemia, ischemic heart disease, stroke : NS
Chatterjee et al., 2023 (36)	AIBL cohort (Australia)	Total: 225, age 74.2 (7.2), 50.7% ♀, APOE ε4: 46.2% AD Aβ+: n=46, age: 75.2 (7.2), 56.5% ♀, APOE ε4: 78.3%	Age, sex, APOE ε4, BMI	Plasma Aβ42/40 ratio, p-tau181, GFAP, NfL [Simoa]	• Age: + p-tau181, GFAP, NfL (only AD) • Female ↑ GFAP (except AD group) • APOE ε4 allele: NS • BMI: ↓ p-tau181, GFAP, & NfL
Dittrich et al., 2023 (78)	H70 Birth Cohort Study (Sweden)	Total 744, age 70 (70–71). 668 control: 50% ♀, 34% APOE ε4 76 CKD: 61% ♀, 25% APOE ε4	eGFR, health condition	Plasma NfL [Simoa]	• eGFR: - NfL • CKD: ↑ NfL
Estepp et al., 2023 (42)	UKADRC cohort (US)	Total 237, age: 82.6, 63% ♀, 19% APOE ε4	Age, sex, APOE ε4	Plasma t-tau, NfL Aβ42, Aβ40, Aβ42/40, [Simoa]	• Age: + Aβ40, NfL; − Aβ42/40, t-tau • Sex: NS • APOE ε4 allele: ↓ Aβ42/40 • Hypertension: ↑ Aβ40, t-tau
Ferreira et al., 2023 (43)	MYHAT study (US)	Total 847, age: 74 [69, 83], 63.9% ♀, 95.5% white, 21.1% APOE ε4 712, cognitively normal, 125 MCI, 10 Dementia	Age, sex, education, APOE ε4	Plasma p-tau181, NfL, GFAP, Aβ42/40 ratio [Simoa]	• Age: -Aβ40, Aβ42, + Aβ42/Aβ40, p-tau181, GFAP & NfL • Female ↑Aβ42/40, GFAP & NfL, ↓ p-tau181 • APOE ε4 allele: ↓Aβ42, Aβ42/40 & NfL • Education: ↓ NfL, GFAP & p-tau181
Janelidze et al., 2023 (56)	BioFINDER (Sweden)	Cohort 1: 141 MCI, age: 73 [65 79], 58.2% ♀, 56% APOE ε4 ; Cohort 2 : total 332 (160 CU, 172 CI), age: 72 [62 77], 51% ♀, 49% APOE ε4	eGFR, health condition	Plasma p-tau181, p-tau217 [IP-MS]	• eGFR: - p-tau181, p-tau217 • CKD: ↑ p-tau181, p-tau217
Ramanan et al., 2023	Mayo Clinic Study of Aging (MCSA) (US)	Total 535, age: 80.2, 69% ♀, 49.9% African American	Sex, ethnicity, diabetes	Plasma Aβ42/40 ratio, p-tau181, GFAP [Simoa]	• Female : ↑ GFAP, ↓ p-tau181 • Ethnicity : NS • Diabetes : ↓ GFAP
Syrjanen et al., 2022 (35)	Mayo Clinic Study of Aging (MCSA) (US)	Total 996, age: 76.4(68.3, 81.9). 43.9% ♀, 90% white. 859 controls: age: 75.63 (67.0, 81.0), 44.7% ♀ 137 MCI/dementia: age: 81.44 (75.6, 85.1), 38.7% ♀	Age, sex, APOE ε4, education, BMI & various health factors	Plasma Aβ42, Aβ40, NfL, t-tau, Aβ42/40 ratio [Simoa]	Among controls: • Age: + Aβ42, Aβ40, NfL, t-tau; − Aβ42/40 ratio • Female ↑ t-tau, ↓ Aβ42 • APOE ε4 allele: ↓ Aβ42 • BMI: ↑Aβ42, Aβ40, t-tau, ↓ NfL • Diabetes: ↑ Aβ42, Aβ40, t-tau • CKD: ↑Aβ42, Aβ40, Aβ42/40, NfL • Hypertension: ↑Aβ42, Aβ40, Aβ42/40, t-tau • Atrial fibrillation: ↑ Aβ40, NfL, t-tau • Chemotherapy: ↑Aβ40 • Previous cancer: ↑ Aβ42/40 & NfL • History of stroke: ↑ NfL & t-tau • Education, head trauma, smoking, depression, anxiety, dyslipidemia : all NS
Yakoub et al., 2023 (45)	PREVENT-AD study (Canada)	Total 373, age: 63.6 (5.1), 72% ♀, 38.6% APOE ε4, 62% Aβ+	Age, sex, APOE ε4, education	Plasma Aβ42/40 ratio, p-tau181, p-tau231, NfL, GFAP [Simoa]	• Age: + NfL, GFAP • Female ↑ GFAP • APOE ε4 allele: ↑ p-tau 231, ↑ GFAP • Education: NS
Yang et al., 2023 (46)	Neurodegeneration and Translational Neuroscience (US)	Total 144. 46 controls, amyloid -: age: 70.7±6.5, 54% ♀, 87% white, 28% APOE ε4 ; 11 control, amyloid +: age: 72.0±6.4, 36% ♀,100% white, 45% APOE ε4 ; 26 CI amyloid - :age: 71.5±6.8, 42% ♀, 92% white, 16% APOE carrier; 61 CI amyloid +:age: 74.2±6.4, 43% ♀, 90% white, 78% APOE ε4	Age, sex, education	Plasma GFAP, p-tau181, NfL [Simoa]	• Age: + NfL, GFAP, p-tau181 • Female ↑ GFAP, p-tau181 (amyloid -) • APOE ε4 allele, education: NS
Zhang et al., 2023 (47)	ADNI cohort, non-randomized cohort study (US and Canada)	Total 645, eGFR &lt; 60 (n = 112): age: 78.0 (9.9), 50.9% ♀, 0.9% memory concerns, 28.6% early-MCI, 21.4% late-MCI, 15.2% AD	Renal function (eGFR),	Plasma Aβ42/40, p-tau181, NfL [Simoa]	• eGFR : - p-tau • within eGFR groups : - with NfL • varied by Aβ-PET status
Hayden et al., 2024 (48)	Look AHEAD RCT (US)	Total 779 (383 DSE and 396 ILI), age: 61.4 ± 6.2, 56.2% ♀, 16.7% African American, 66.2% White, 22.8% APOE ε4	Age, sex, APOE ε4, BMI, education, eGFR, ethnicity, lifestyle factors, health conditions	Plasma Aβ42, Aβ40, Aβ42/40, p-tau181, NfL, GFAP [Simoa]	• Age: + Aβ40, Aβ42, NfL, GFAP • Female ↓ Aβ40, NfL, ↑ GFAP • APOE ε4 allele: ↓ Aβ42/40 • BMI: − GFAP • Education: + GFAP • eGFR: + Aβ42, Aβ40, – Aβ42/40, NfL • Ethnicity: Hispanic and Black ↓ Aβ40, ↑Aβ42/40, ↓ NfL • Alcohol, history of CVD: ↑ GFAP • Diabetes: ↑Aβ42/40, ↑ NfL • Hypertension: ↑ Aβ42, Aβ 40 • Depression : ↓ GFAP • HbA1c : - Aβ42, Aβ40 • Smoking, Dyslipidemia : NS
Sarto et al., 2024 (44)	Alzheimer’s disease and other cognitive disorders Hospital Clinic (Spain)	Total 360 (36 without impairment, 67 SND, 174 AD, 21, LBD and 62 FTD), age: 66.5 (7.7), 55% ♀	Age, sex, APOE ε4, BMI, eGFR, chronic conditions	Plasma p-tau181, GFAP and NfL [Simoa]	• Age: + p-tau181, GFAP, NfL • Female & APOE ε4 allele :↑ p-tau181, GFAP • BMI & eGFR: ↓ p-tau181, GFAP, NfL • CKD: ↓ NfL • Blood volume: - NfL • Smoking, hypertension, diabetes, cancer, MI: all NS
Wu et al., 2024 (52)	Shanghai Aging Study (China)	Total 1189, age: 70.24 [64.32 75.88], 54.2% ♀, 17% APOE ε4, all without dementia	eGFR	Plasma p-tau181, NfL [Simoa]	• eGFR: - NfL, p-tau181

Abbreviations: Aβ: amyloid beta; AD: Alzheimer’s disease; ADNI: Alzheimer’s Disease Neuroimaging Initiative; AIBL: Australian Imaging, Biomarker & Lifestyle Flagship Study of Ageing; ALSPAC: Avon Longitudinal Study of Parents and Children; APOE-ε4: apolipoprotein E allele; APP: amyloid-β precursor protein; BMI: body mass index; BP: blood pressure; CI: cognitively impaired; CKD: chronic kidney disease; eGFR: estimated Glomerular Filtration Rate; FALT: Functional Assessment in Liver Transplantation study; FRS: Framingham Risk Score; GFAP: glial fibrillary acidic protein; HABS-HD: Health& Aging Brain study among Latino Elders, HABLE study; IP-MS: immunoprecipitation mass spectrometry; KARVIAH: The Kerr Anglican Retirement Village Initiative in Ageing Health cohort; LDL-C: serum low-density lipoprotein; MCI: mild cognitive impairment; MCSA: Mayo Clinic Study of Aging; MYHAT: Monongahela-Youghiogheny Healthy Aging Team; NAFLD: non-alcoholic fatty liver disease; NCGG: National Center for Geriatrics and Gerontology; NfL: neurofilament light chain; PET: positron emission tomography; PREVENT-AD: Pre-symptomatic Evaluation of Experimental or Novel Treatments for Alzheimer’s Disease; p-tau: phosphorylated Tau; TC: serum total cholesterol; t-tau: total Tau; UKADRC: University of Kentucky Alzheimer’s Disease Research Center.

The majority of studies assessed whether biomarker levels varied according to age and/or sex (69% and 66%, respectively). The most consistent finding was a significant positive association between NfL and age, reported in all studies that measured NfL. These studies included both cognitively unimpaired individuals, as well as individuals with cognitive impairment and dementia. Likewise, for GFAP all but two studies reported a positive correlation with age. In contrast, some studies of p-tau reported a significant positive association with age, whereas others reported no association. The findings regarding Aβ were less consistent. For example, seven studies reported no significant association between Aβ42/40 ratio and age, but four reported negative associations and two other studies reported positive associations. In terms of sex, the majority of studies reported that NfL and Aβ42/40 ratio did not vary by sex (12 and 11 studies, respectively), but multiple studies found that females had higher GFAP than males (7 studies). Four studies found that p-tau181 did not differ by sex, while three studies reported that females had higher levels and another two studies reported higher levels in men (38, 43).

A total of 14 studies examined whether the presence of the APOE-ε4 risk allele was significantly associated with plasma AD biomarker levels. The most consistent findings were a significantly lower Aβ42 (4 studies, 75%) and Aβ42/40 ratio (5 studies, 67%). In addition, there were no significant association with NfL (8 studies, 89%) and p-tau181 (6 studies, 67%). Only one study reported a lower level of NfL and 3 studies reported a higher level of p-tau181 among APOE-ε4 carriers. Only five studies assessed the level of plasma biomarkers in different racial groups. Findings suggested lower Aβ42/40 ratio and t-tau, and higher NfL in whites compared to other racial or ethnic groups (37, 40, 48), whereas other studies did not report any significant associations.

Eight studies examined the variation in levels of plasma biomarkers by BMI. Most of these studies reported a significant negative association with GFAP (67%) and NfL (75%), and to a lesser extent with p-tau (50%), but no association with Aβ42/40 ratio (67%). Apart from education level examined by 9 studies with mixed findings, other lifestyle exposures were less commonly examined, including physical activity (2 studies), smoking (4 studies) and alcohol consumption (2 studies).

In terms of chronic health conditions, the most frequently investigated has been kidney function using chronic kidney disease (CKD, 5 studies), or estimated Glomerular Filtration Rate (eGFR, 7 studies) or creatinine (2 studies), which are indicators of kidney function. The vast majority of these studies found that individuals with CKD, compared to those without, had higher biomarker levels; similarly, worse kidney function was associated with higher biomarker levels. This was found in all studies investigating NfL, Aβ42 and Aβ40, and in 80% of studies looking at p-tau181 and/or t-tau. In contrast, there was no consistent findings with regards to CKD and levels of the Aβ42/40 ratio. One study reported higher Aβ42/40 ratio in individuals with CKD (35), another no significant association (40), and eGFR was negatively associated with Aβ42/40 ratio in one study (48), but positively associated in another (39).

In terms of other chronic conditions, hypertension (8 studies) and diabetes (8 studies) have been the most frequently examined. These chronic conditions have been associated with increased Aβ42 (88%) and Aβ40 (100% of studies), but they were not associated with GFAP, p-tau181, nor Aβ42/40 ratio; and only 50% of studies reported diabetes was associated with increased NfL. The majority of other chronic conditions have only been examined by a small number of studies, and of those, few have reported significant associations.

## Discussion

As a simple, accessible, and potentially scalable high-throughput test, plasma biomarkers of AD and neurodegeneration could provide the opportunity to identify individuals in the preclinical phase before dementia symptoms are apparent, potentially transforming the diagnosis of AD dementia. Blood biomarkers could thus enable interventions to begin prior to the onset of symptoms, at a stage when minimal neurodegeneration has occurred and treatments are most likely to be beneficial in slowing disease progression. However, as these biomarkers near clinical use, it is essential to understand what factors could affect the levels of these blood biomarkers, and thus need to be considered before reference ranges for these biomarkers are developed.

This review summarises existing evidence for an association between sociodemographic and lifestyle factors, and chronic conditions, on plasma biomarkers of AD and neurodegeneration. Overall, this evidence suggests that older age is associated with higher NfL and GFAP, and females had higher GFAP levels than men, and possibly higher t-tau. Carriers of the APOE-ε4 allele were frequently found to have lower Aβ42 and Aβ42/40 ratio, but most studies of these studies reported no association with p-tau181 and NfL. Higher BMI was generally associated with lower GFAP, NfL and p-tau, and better kidney function with lower NfL, p-tau181, t-tau, Aβ42 and Aβ40. Together these findings highlight the importance of considering factors such as age, sex, and health conditions when interpreting blood biomarker levels, in the context of AD diagnosis and predicting future risk, but further research is needed.

Research indicates that CKD is a strong risk factor for cognitive disorders, particularly dementia (49, 50). Similarly, patients with liver disorders appear to have a higher risk of developing AD (23, 51). The kidney and liver play an essential role in the clearance of circulating proteins from the blood. Poor renal and hepatic function can thus impact the peripheral clearance of these AD and neurodegeneration biomarkers (23, 52), resulting in increased levels in the blood. This is supported by the findings of studies that demonstrate elevated levels of these biomarkers in individuals with CKD (35, 38). While there have not many studies which have looked at liver dysfunction, the two studies that examined the effect of hepatic function also reported higher plasma biomarker levels (22, 24). Physiological processes can also account for the association between higher BMI and lower levels of plasma biomarkers. Higher BMI is linker with a greater blood volume (53, 54), which in turn, can dilute the overall concentrations of circulating proteins. Indeed, in a study of morbidly obese individuals who had lower concentrations of circulating NfL and GFAP compared to lean individuals, found that following bariatric surgery-induced weight loss, circulating levels increased (55). Ignoring factors which influence physiological processes when interpreting blood biomarker levels, could thus led to false positives or negative dementia diagnosis. Indeed, it has been shown that the difference in plasma biomarker levels (p-tau181) between participants with and without CKD exceeded the differences between biomarker levels between participants that were amyloid PET positive versus negative (38). Levels of the Aβ42/40 ratio appeared to be more robust, and less commonly affected by these physiological processes (41, 47), and examining the ratio of p-tau to t-tau may also be more beneficial in this regard (56).

The extent to which other chronic conditions could impact circulating biomarker levels is unclear. For example, the liver plays a central role in metabolic detoxification and the expression of metabolic enzymes is impaired in cirrhosis, as well as obesity and diabetes. The impact of having both cirrhosis and obesity on biomarker levels is unclear. Furthermore, several drugs can affect the production, clearance, or metabolism of proteins in the body, and thus could also lead to alterations in biomarker levels. To our knowledge, only one study has looked extensively at different medications, and found that a number of specific medications, were significant contributors to the variance in plasma biomarker levels (57). A recent study reported that sacubitril/ valsartan which is used for heart failure, was associated with increased Aβ40 and Aβ42, and a reduction in Aβ42/40 ratio (58). This finding aligns with the known pharmacology of this drug, which inhibits neprilysin that is involved in clearing Aβ (59). More research is needed to better understand the potential influence of chronic health conditions, as well as drug treatments, on plasma biomarkers. Given that chronic conditions become increasing commonly as people age, and that older adults are those most susceptible to being screened for AD, this emphasizes the importance of more work in this area.

Other factors may influence measured biomarker levels because of physiological processes, as well as potentially direct impacts on neuropathological burden. The presence of the APOE-ε4 allele increases amyloidosis (21) and is the strongest genetic risk factor for late onset AD (60). This aligns with the finding that ε4 carriers were found to have lower Aβ42/40 ratio. The increase in NfL and GFAP with age, aligns with age being the strongest risk factor for neurodegeneration and results from an accumulation of cellular and molecular damage over time. However current clinically available NfL tests do not have age-specific cut-offs. Age is also a strong risk factor for AD, but not all studies have found age to be associated with an AD “risk profile” (i.e. characterised by higher tau and lower Aβ42 and Aβ42/40 ratio) (61). While Mielke et al., reported a significant positive association between age and both p-tau181 and p-tau217, they also found that this association was mainly driven by amyloid PET positive individuals (40). This indicates that p-tau might not increase with age among individuals without brain amyloid deposition.

Sex differences in plasma biomarkers have not been consistently reported, but there are several ways in which sex could affect the concentration or interpretation of blood biomarkers. Females appear to have higher blood-brain permeability than males (62), which could result in higher concentrations of brain-derived biomarkers in the blood for males. Sex differences in platelet activation (63) and blood protein concentrations could also affect the measurement (64) or levels of Aβ in the blood (65). Furthermore, differences in comorbidities between males and females (e.g. kidney function) (66) could influence sex differences in the clearance and measurement of these biomarkers. For example, one study found that males have higher levels of p-tau181 and p-tau217 (38), which contrasts with the findings of some other studies (44, 46). However, this difference was attenuated after excluding individuals with chronic kidney disease (38).

Dementia disproportionately impacts Blacks (67), who are 2–3 times more likely to develop dementia than whites (68, 69). There may be ethnic and racial differences in AD plasma biomarkers, however to date, there has been a substantial lack of diversity in these studies. A systematic review of AD fluid biomarkers levels between Blacks and whites found only one study which had measured these biomarkers in plasma (70). Of the studies included in this review, only a couple looked at possible differences based on race or ethnicity (37, 40, 48). Compared to non-Hispanic whites, higher Aβ42/40 ratio was found in Mexican Americans, Hispanics and Blacks, but lower NfL in Mexican Americans and Blacks (37, 40, 48). It should also be noted that the prevalence of many health conditions may differ by sex and ethnicity (71), and these factors may also contribute to observed sex and ethnic differences in biomarker levels.

There are limitations to this review which need to be considered. Firstly, it was a scoping review of the literature rather than a systematic review, and thus was not intended to include every study that has been published on this topic. However, a systematic search was used to capture relevant studies, and the extracted data was verified by two co-authors. The studies included in this review examined a broad range of factors, and in varying populations across different countries. It is possible however, that some factors have been examined and were not included in this review. Further, it is well recognised that individuals who volunteer to participate in research studies are not fully representative of the broader population. They are more likely to be highly educated, female and white (72). This limits the extent to which these findings can be generalised to other populations. The relatively small size of many studies included in this review mean they were under-powered to detect moderate to small effect sizes, and this could explain some of the inconsistent findings (73). Most conditions, have been inadequately studied to date, and differences in study populations, their age and cognitive status (e.g. normal, mild cognitive impairment (MCI), dementia) is also likely to influence the findings. For example, Syrjanen et al. found that only amongst controls (but not participants with MCI or dementia), women had higher t-tau levels and a lower Aβ42/40 ratio than men (35). A preliminary study involving 177 individuals showed that diet was associated with plasma biomarker levels (74). Increased intake of both ‘healthy’ and poor food choices (e.g. grains and beans, as well as fast food and red meat) was associated with increased NfL, and higher olive oil consumption was associated with lower p-tau181 (74). However, the Aβ42/40 ratio was not associated with specific foods (74). Larger studies which include a wide range of factors are needed. Finally, it is important that researchers consider the potential for confounding when measuring plasma biomarkers, and account for this appropriately. This includes firstly recognizing and capturing information on potential confounders and then adjusting for these through multivariate methods or stratification.

## Conclusion

Blood biomarkers are relatively feasible and inexpensive to measure, and may enhance AD diagnosis in the primary care setting. However, it is critical to first understand how factors can affect the interpretation of these biomarkers, through an effect on physiological processes (e.g. peripheral clearance, blood volume), or amyloid pathology and neuropathological burden directly (e.g. APOE ε4), being established risk factors for AD. Some factors may influence biomarker levels through a combination of these processes. For example, chronic kidney disease could be a risk factor for dementia, but also affect renal clearance of biomarkers from the blood (75, 76). Understanding the aetiology and impact of sociodemographic, lifestyle and health factors on blood biomarkers will be essential for the establishment of reference ranges and thus the correct interpretation of these biomarkers in the clinical setting.
